# Postmortem Confirmation of Human Rabies Source

**DOI:** 10.3201/eid1205.051425

**Published:** 2006-05

**Authors:** Rafael Oliveira, Neide Takaoka, Paulo Brandao, Pedro Carnieli, Carla Macedo, Juliana Castilho, Maria Luiza Carrieri, Ivanete Kotait

**Affiliations:** * Instituto Pasteur, São Paulo, Brazil

**Keywords:** human rabies, exhumation, fluorescent antibody test (FAT)

**To the Editor:** Rabies is a fatal encephalitis caused by a neurotropic RNA virus of the family *Rhabdoviridae*, genus *Lyssavirus*. The predominant rabies virus reservoir hosts are bats and carnivores. Among these, rabid dogs represent a substantial public health problem, particularly in developing countries (*1*).

Laboratory diagnosis of rabies is essential to guide control programs, epidemiologic surveys, and prophylactic measures (*2*). Among the laboratory tests recommended by the World Health Organization (WHO), the fluorescent antibody test (FAT) is the accepted standard for rabies diagnosis (*1*). Although rabies virus antigens can be detected in decomposed samples, FAT is less effective when such samples are tested. In those cases, polymerase chain reaction (PCR) can provide better results (*3*). Since the degree of decomposition at which FAT starts to become ineffective is unknown (*4*), when smears from decomposed samples are made for FAT, a suspension of the same brain tissues should be made in the appropriate diluents for the mouse inoculation test (MIT), cell culture, or reverse transcription–polymerase chain reaction (RT-PCR) (*2*). However, if all test results are negative, rabies cannot be ruled out because of the condition of the sample.

On February 28, in the city of Carbonita, Minas Gerais State, in southeastern Brazil, a 62-year-old man was bitten by a bat on the right ankle. Approximately 50 days later, his leg began to feel numb, and he experienced a continuous headache, pain at the site of the bite, convulsions, frequent urge to clear his throat, hiccups, nausea, difficulty in swallowing, dry lips, slightly elevated body temperature (37°C–37.5°C), paralysis of superior and inferior left limbs, shaking, and hallucinations. On May 4, 16 days after clinical manifestations began, the patient died; the cause of death was registered as a cerebral vascular accident. One month later, the body was exhumed to obtain a sample from the central nervous system (CNS), which was sent to Instituto Pasteur, São Paulo, registered as sample 5341 M/04 and tested by FAT, MIT, and RT-PCR.

In total, 8 smears were prepared from the sample to be analyzed by FAT according to the method of Dean et al. (*5*) with fluorescein isothiocyanate–labeled polyclonal antinucleocapsid antibodies. MIT was carried out as described by Koprowski (*6*) with 7 mice. For RT-PCR, RNA was extracted from the CNS sample with TRIzol, according to the manufacturer's instructions (Invitrogen, Rockville, MD, USA). RT-PCR was carried out with modifications as described by Orciari et al. (*7*), with primers 504 (sense) and 304 (antisense), aiming at the amplification of a 249-bp fragment of rabies virus nucleoprotein (N) gene, by using Superscript II (Invitrogen) and Taq DNA-polymerase (Invitrogen).

Fluorescent inclusions were observed in 6 of the 8 slides prepared for the FAT. The RT-PCR of the RNA sample resulted in amplicons of the correct size (249 bp), as did the positive control sample, CVS strain rabies virus. No bands were observed in the reaction corresponding to the negative/reagent control (ultra-pure water). The MIT results were negative. Because the virus could not be isolated, antigenic typing with monoclonal antibodies could not be performed.

The fragment obtained in the RT-PCR was bidirectionally sequenced with DYEnamic ET Dye Terminator (Amersham Biosciences, Piscataway, NJ, USA) in a MegaBACE DNA sequencer (Amersham Biosciences) and resulted in a 165-nucleotide sequence. The final sequence was aligned with homologous sequences from GenBank by using the ClustalW (available from http://www.ebi.ac.uk/clustalw) and Bioedit software (Isis Pharmaceuticals, Carlsbad, CA, USA). The phylogenetic tree was produced by using the neighbor-joining DNA-distance method and the Kimura 2-parameter model with 1,000 bootstrap replicates in Mega 2.1 (version 2.1) (available from http://www.megasoftware.net/). The sequence was segregated in the variant 3 cluster (*Desmodus rotundus*–related variants), which suggests that *D*. *rotundus* is the most probable source of infection (Figure). The sequence was assigned GenBank accession no. DQ177278.

**Figure:**
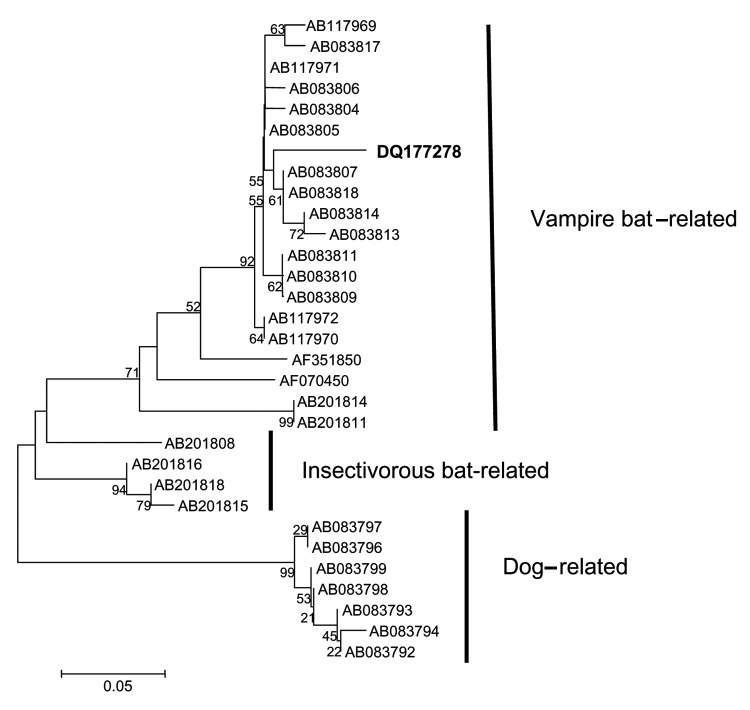
Neighbor-joining phylogenetic tree to a stretch of the 3´ end of the N gene of rabies virus variants related to vampire bats, insectivorous bats, and dogs. Strain DQ177278 is shown in **bold**. The bar indicates the genetic distance scale. Numbers at each node indicate 1,000 replicates of bootstrap values.

The lack of diagnosis or delay in diagnosis can increase the number of persons potentially exposed to rabies virus infection by contact with the patient or even by organ transplantations (*8*). Moreover, an early diagnosis can decrease the cost of treatment by eliminating the use of ineffective drugs and unnecessary diagnostic tests (*2*), as well as allowing potentially useful emerging therapeutic strategies to be used (*9*).

Before this report, no reference of a rabies diagnosis by FAT or RT-PCR had been reported from a human exhumed 30 days postmortem. The RT-PCR results agree with those obtained by David et al. (*10*) from a decomposed sample of animal origin after 36 days.

These facts demonstrate that rabies should be considered in cases of encephalitis with the classic clinical signs and symptoms as well as the paralytic form of disease (paresis and paralysis). Rabies should be suspected when early clinical symptoms, for example, itching and paresthesia, are demonstrated at the local site of infection. In addition, the laboratory investigation showed that molecular methods such as RT-PCR and sequencing were sensitive assays for nucleic acid detection and determination of the rabies virus variant in this unusual case from an exhumed human.
